# Nipple Adenoma: Case Report of a Rare Entity

**DOI:** 10.7759/cureus.22996

**Published:** 2022-03-09

**Authors:** Yagnya D Dalal, Aditya K Trivedi, Viraj Panchal, Yatri Patel, Darshit D Dalal

**Affiliations:** 1 Surgery, Gujarat Cancer Society (GCS) Medical College, Ahmedabad, IND; 2 Surgery, Ahmedabad Municipal Corporation's Medical Education Trust (AMCMET) Medical College, Ahmedabad, IND; 3 Surgery, Smt. Nathiba Hargovandas Lakhmichand (NHL) Municipal Medical College, Ahmedabad, IND; 4 Surgical Oncology, Cancer Care Centre, Ahmedabad, IND

**Keywords:** myoepithelial cells, lactiferous duct, erosion, paget’s disease, nipple adenoma

## Abstract

A nipple adenoma is a rare benign breast tumor. The commonest presentation of this rare entity is nipple erosion, serosanguinous discharge, induration, or tumor formation at the nipple. It often mimics malignant breast lesions or nipple eczema and is mistaken for Paget’s disease of the nipple or dermatological pathology. It may be misdiagnosed pathologically as ductal carcinoma of the breast. This may cause a diagnostic delay or a faulty diagnosis. Treatment is the excision of the tumor with or without nipple excision. Here, we report a case of nipple adenoma that projected out of the nipple along with nipple erosion, serosanguinous discharge, and occasional bleeding from the adenoma. A 37- year-old woman presented with a tumor on her right nipple for eight months, with the erosion of the nipple and serosanguinous discharge. The patient gave a history of a small amount of bleeding occasionally. Axilla was normal. The patient was advised to have a mammosonography. It showed an oval-shaped, well-demarcated, hypoechoic, uniformly solid nodule in the right nipple. There was no microcalcification seen on mammography. A punch biopsy was done to establish the diagnosis. It showed ductal hyperplasia and papillary proliferation of glandular structures suggestive of nipple adenoma. Complete resection of the tumor with partial excision of the nipple was done with a satisfactory cosmetic result. Though very uncommon, the possibility of nipple adenoma should be thought of when a patient presents with nipple erosion and discharge with or without a clinically obvious tumor. Timely diagnosis with histopathological correlation is important since it allows for less invasive surgical methods. In our case, we could attain a cosmetically satisfactory outcome without a remnant tumor. Paget’s disease of the nipple also has a similar clinical presentation, and it is a premalignant condition. The objective of presenting this case is to highlight the possibility of this rare benign condition, which may be easily missed clinically and also demands careful histopathological examination for its correct diagnosis.

## Introduction

Nipple adenomas are benign and rare epithelial proliferations that arise from the lactiferous ducts of the nipple. They are also known as "erosive adenomas" or "subareolar adenomas" [1]. They commonly present as a smooth nodule on the nipple or nipple erosion with discharge, nipple deformity, and ulceration. A clinical diagnosis can be challenging and histological confirmation is essential to exclude other pathologies like Paget’s disease of the nipple or breast malignancy [2-4]. The lesion is most commonly present in females in their fourth decade of life but has been reported in men, adolescents, and infants [5,6]. Nipple adenomas are relatively small benign lesions ranging from 0.5 to 3.5 cm [1,7]. Though benign, it can locally infiltrate smooth muscle and nerves within the nipple stroma but does not metastasize. As this condition is very rare, most of the literature is in the form of case reports or case series.

## Case presentation

**Figure 1:**
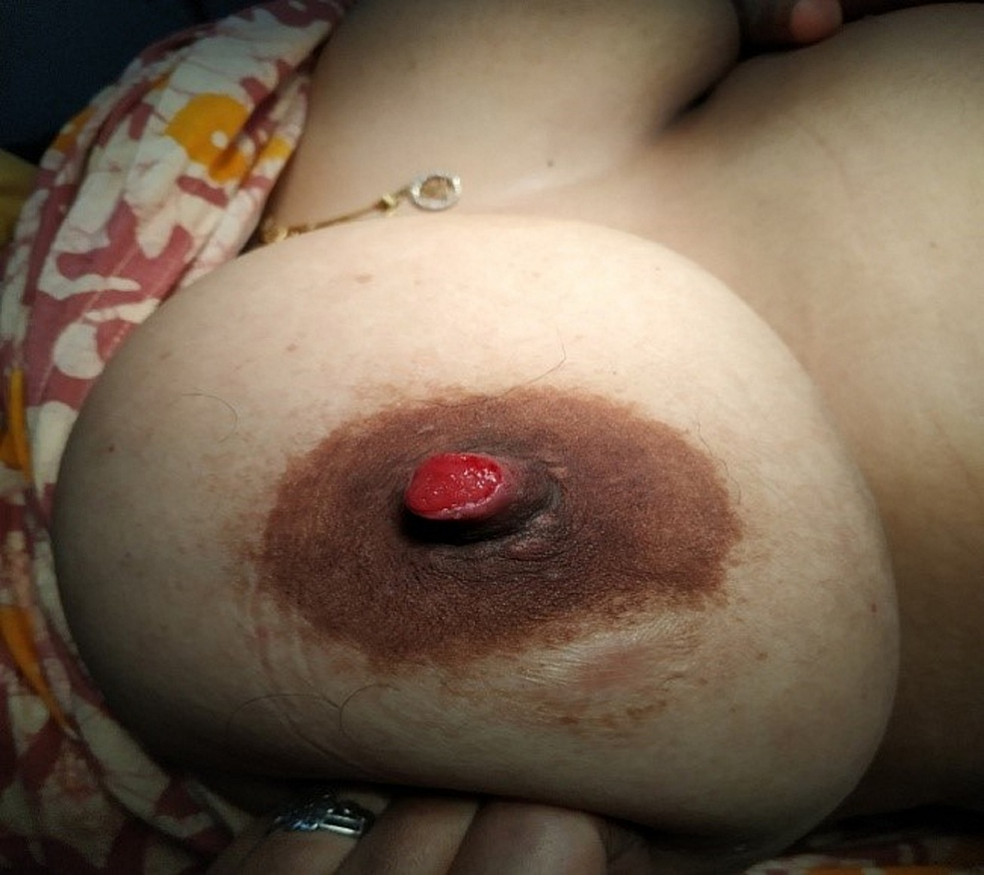
Right nipple adenoma seen projecting out of the nipple. Nipple erosion is evident. Tumor is bright red in color with granulation-like texture.

**Figure 2:**
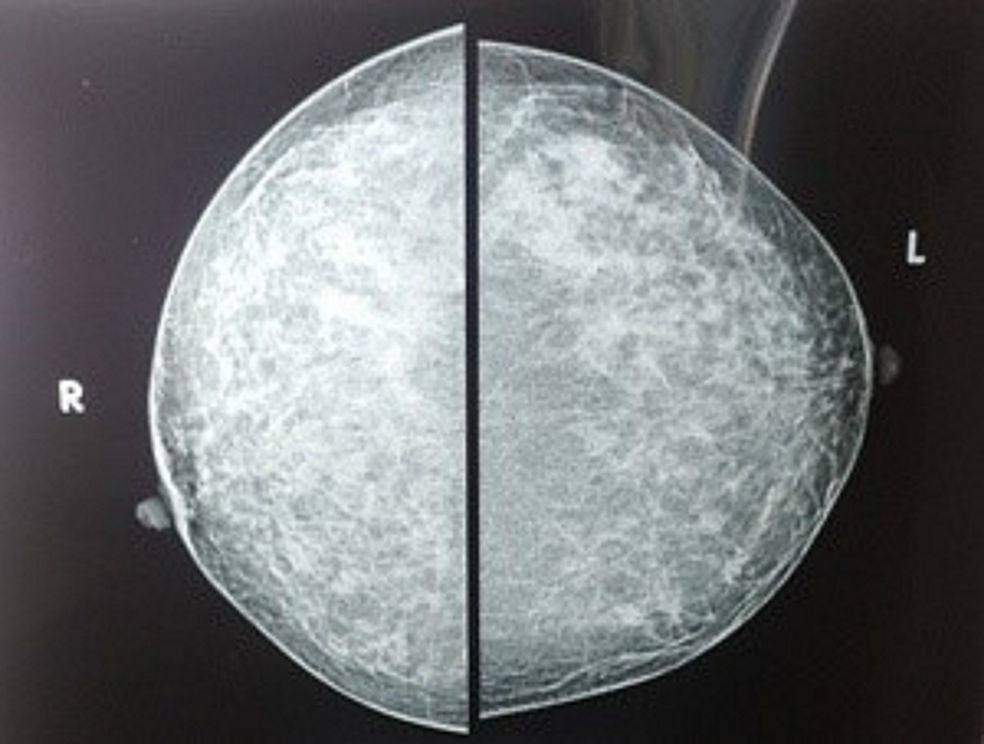
Oval shaped, well demarcated, uniformly solid nodule without microcalcification on mammography.

**Figure 3:**
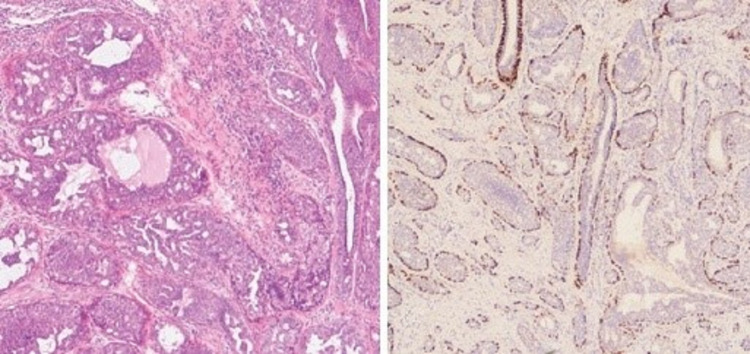
(a) Ductal hyperplasia and papillary proliferation of glandular stroma and (b) intact myoepithelium on p63 staining.

A 37-year-old woman presented with a red-colored tumor projecting through her right nipple for five months. It began as an erosion of the nipple with serosanguinous discharge for the initial two months, followed by the development of a soft, fragile tumor projecting out through the nipple (Figure 1). A history of mild bleeding on friction with clothes was present. She had two living children. Both were delivered at full term via cesarean section. Both were breastfed for one year. She had never used any pharmacological contraceptives or any kind of hormone therapy. Her age of menarche was 13 years. She had regular menstrual cycles without any premenstrual symptoms. She was not addicted to tobacco or alcohol. There was no history of any breast or ovarian pathologies in her first-degree maternal relatives. On clinical examination, the tumor was seen projecting out of the right nipple with the erosion of the overlying part of the nipple. It was a cherry-red, granulation-like lesion. Serosanguinous discharge was present. Mild bleeding on touch was present. The tumor was palpable through the whole length of the nipple up to its base. Both the axillae and left breast were normal clinically. She consulted her family physician about the same and was advised to take daily dressings and some oral antibiotics for two weeks. She underwent regular dressing thereafter for about four months. There was no improvement in her condition, so mammosonography was advised by her family physician. It revealed a well-demarcated hypoechoic solid nodule with uniform density in the right nipple without microcalcification (Figure 2). Both the axillae and left breast were unremarkable on mammosonography. She was referred to the oncosurgeon. A punch biopsy was done by an oncosurgeon. It showed ductal hyperplasia and papillary proliferation of glandular stroma suggestive of nipple adenoma (Figure 3). Immunohistochemistry with p63 was carried out to confirm the diagnosis. It showed intact myoepithelium. The diagnosis of nipple adenoma was confirmed by histopathological and immunohistochemical analysis.

**Figure 4:**
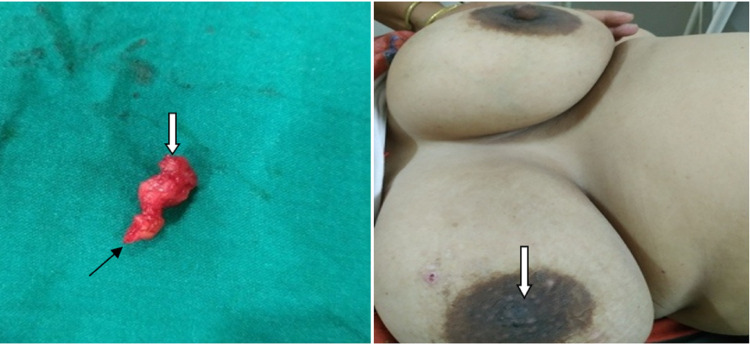
(a) Excised tumor. Black arrow indicates tumor part at nipple base. White arrow indicates distal most protruding part of tumor. (b) Tenth postoperative day of reconstructed nipple indicated.

The patient was operated on under general anesthesia. A nipple-splitting incision was placed at the 3 o’clock position, extending beyond the nipple-areola junction. The tumor was excised completely. Around 4 mm of the distal-most part of the nipple was excised because it was edematous and chronically inflamed. The nipple was reconstructed with a satisfactory cosmetic result (Figure 4). The patient regularly comes for follow-up, and she is absolutely fine.

## Discussion

Nipple adenomas are a rare benign pathology, with most breast units expecting to see one to two cases per year [1]. It was first described by Jones [2]. It occurs most often in middle-aged females [2,8,9]. Clinically, it presents as nipple discharge, nipple erosion, or ulceration on the nipple. A tumor may be palpable below the nipple [8]. Our patient had a palpable tumor from the base of her right nipple that continued through the nipple and projected out of it. It had a red granulation-like texture. According to the World Health Organization (WHO) classification of breast tumors established in 2003 [2,9], an adenoma of the nipple is defined as "a compact proliferation of small tubules lined by epithelial and myoepithelial cells, with or without proliferation of the epithelial component, around the collecting ducts of the nipple" [10]. The Japanese Breast Cancer Society defined the nipple adenoma as "a tumor developing papillary or solidly in the lactiferous duct of the nipple or just under the areola" [3]. Nipple adenomas may be locally infiltrative. Surgical excision with clear margins is the treatment of choice. The histopathological analysis is very important to confirm the diagnosis and confirm clear margins after the tumor's resection. Histological findings include a well-circumscribed tumor with ductal hyperplasia, the papillary proliferation of glandular stroma, and the lining of glandular and tubular structures with an inner layer of epithelial cells and an outer layer of myoepithelial cells [3,11,12]. Mitoses may be seen, but cellular atypia is absent [3,11,12]. Nipple adenoma may be misdiagnosed as ductal carcinoma, especially when there is the presence of marked proliferative growth of intraductal tumor cells. The histologically most important finding that distinguishes nipple adenoma from ductal carcinoma is the presence of two distinct layers of myoepithelial cells in lactiferous ducts [2,10,13,14]. The presence of myoepithelial cells in the lactiferous ducts can be confirmed by immunohistochemical staining using p63, α-smooth muscle actin, or CD 10 [10,13]. Since the tissue density of nipple adenoma is similar to that of normal breast tissue, it may not be detected by conventional imaging modalities of the breast [12,13]. Breast magnetic resonance imaging (MRI) may be used to assess the extent of the tumor's involvement [8,15]. However, the pattern of nipple adenoma may be confused with breast malignancy on MRI [16]. A confirmed diagnosis can only be made by histopathological analysis and immunohistochemistry. Accurate and timely diagnosis avoids unnecessary extensive surgery and psycho-trauma for the patient. Minimally invasive procedures like Mohs micrographic surgery or cryosurgery have a definite role in the management of early-detected cases [16]. Nipple adenoma will continue to enlarge and cause local destruction of the nipple if left untreated [16]. In our case, we could perform complete resection of the adenoma through a nipple splitting incision with partial excision of the distal-most part of the nipple. Cosmetic restoration of the nipple was satisfactory. The patient does not have any complaints after six months of follow-up.

## Conclusions

Nipple adenoma is a very rare benign tumor that clinically mimics Paget’s disease of the nipple or malignant breast lesions. Because of the rarity of its occurrence, nipple adenoma may be easily missed as one of the differentials in clinical diagnosis. The possibility of nipple adenoma should be thought of when a patient presents with nipple erosion and discharge with or without a palpable tumor beneath the nipple. It also poses a challenge for histological diagnosis. Accurate histological and immunohistochemical analysis is essential to distinguish nipple adenoma from invasive carcinoma. Timely diagnosis with proper histopathological analysis is vital since it allows for less invasive surgical methods. In our case, we could achieve a cosmetically satisfactory outcome without the remnant tumor.
